# Detection of Salient Crowd Motion Based on Repulsive Force Network and Direction Entropy

**DOI:** 10.3390/e21060608

**Published:** 2019-06-20

**Authors:** Xuguang Zhang, Dujun Lin, Juan Zheng, Xianghong Tang, Yinfeng Fang, Hui Yu

**Affiliations:** 1 School of Communication Engineering, Hangzhou Dianzi University, Hangzhou 310018, China; 2 School of Electrical Engineering, Shandong Huayu University of Technology, Dezhou 253034, China; 3 School of Creative Technologies, University of Portsmouth, Portsmouth PO1 2DJ, UK

**Keywords:** crowd behavior analysis, salient crowd motion detection, repulsive force, direction entropy, node strength

## Abstract

This paper proposes a method for salient crowd motion detection based on direction entropy and a repulsive force network. This work focuses on how to effectively detect salient regions in crowd movement through calculating the crowd vector field and constructing the weighted network using the repulsive force. The interaction force between two particles calculated by the repulsive force formula is used to determine the relationship between these two particles. The network node strength is used as a feature parameter to construct a two-dimensional feature matrix. Furthermore, the entropy of the velocity vector direction is calculated to describe the instability of the crowd movement. Finally, the feature matrix of the repulsive force network and direction entropy are integrated together to detect the salient crowd motion. Experimental results and comparison show that the proposed method can efficiently detect the salient crowd motion.

## 1. Introduction

Video surveillance plays an important role in monitoring crowd safety, which is one of the key concerns in our daily life. Since the traditional human-computer interaction between video surveillance and crowd safety is time-consuming and labor-intensive, intelligent video surveillance issues such as target tracking, target detection and crowd analysis have become popular research topics. Crowd motion detection and analysis are essential for crowd behavior understanding [1,2]. It is thus very important to detect the salient motion in the crowd to monitor any potential threats or even damage to social safety. Salient motion has been defined as motion that is likely to result from a typical surveillance target as opposed to other distracting motions [3]. According to this definition, salient crowd motion usually indicates areas that are inconsistent with the mainstream pedestrians’ movement. For video surveillance, these areas deserve more attention.

In recent years, due to the rapid development of computer vision technologies, progress has been made in detection of crowd saliency. For example, Lim et al. [4,5] proposed a method for automatically detecting a salient region using time variation of a crowd scene flow field by detecting the fluid activity in a given scene and detecting saliency with a minimum amount of observation region. Some methods for detecting globally salient motion regions for spectral singularity analysis of motion regions in video [6,7] have been also presented. Zhou et al. [8] studied the invariance of coherent neighbors as coherent motion priors, and proposed an effective clustering technique to detect crowd saliency. Solmaz et al. [9] overlaid the scene from the particle grid of the dynamic system defined by the optical flow, and proposed a method to identify the behavior of five people in the visual scene through time integration. Zhang et al. [10] surveyed physics-based methods for crowd video analysis and sorted out the existing public database of crowd video analysis. Although many methods have shown good performance in crowd salient motion detection, the internal mechanism of crowd movement still needs to be explored. The pattern of crowd movement depends on both individual movement and interaction between individuals. It is of great value to explore a method to describe individual interaction and apply it to crowd salient motion detection. 

**Figure 1:**
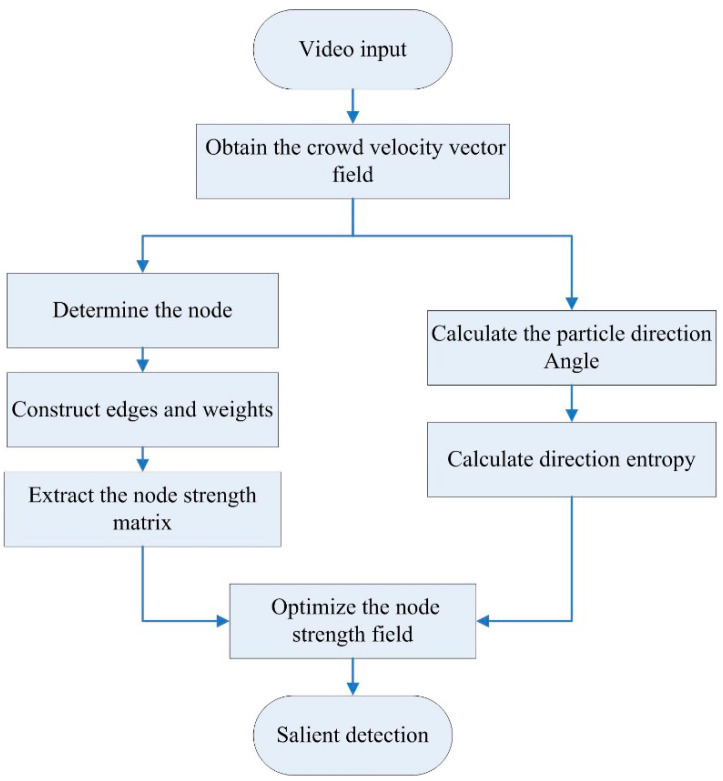
Framework of salient crowd motion detection based on repulsive force network and direction entropy.

In this paper, we propose a salient crowd motion detection method based on a direction entropy and a repulsive force network. The optical flow is first obtained using the pyramid-based Lucas-Kanade optical flow algorithm. Then, the weighted network is constructed by the repulsive force and the node strength matrix is obtained by using the node degree as the characteristic parameter. Finally, the particle motion direction entropy is used to optimize the node strength matrix and to detect salient movements of the crowds. The framework of the proposed method is shown in Figure 1. A motion vector field is established by giving each pixel a velocity vector in each image through the Pyramid Lucas-Kanade optical flow algorithm. Each vector in the crowd vector field is treated as a moving micro-particle. In order to build a complex network model, we regard each particle and the relationship between two particles as node and edge in the network, respectively. In order to show whether there is a connection between two particle nodes, we use the interaction force to construct the network. After calculating by optical flow method, the position and velocity parameters of each particle can be determined. Whether there is an edge between the nodes depends on the value of repulsive force between these nodes. The repulsive force can be described by the inertial centrifugal force. The value of the inertial centrifugal force is the weight of the edge and a velocity vector node can be selected accordingly. In the neighborhood of the node, the relevancy between the two velocity vectors is taken as a condition to determine the relevancy between the corresponding nodes.

A weighted crowd network model is constructed to obtain the adjacency matrix representing the crowd motion information. In order to obtain a complete boundary of salient motion region, the velocity field is reversed and the repulsive force between particles is calculated repeatedly to construct the repulsive force network model. Then, the edge and weight are constructed by the repulsive force model, and the results of the superposition are taken as a construction step. Once all nodes are traversed, the strength of each crowd-weighted network node is extracted as a characteristic parameter to construct the strength matrix of the nodes. By calculating the direction of the velocity entropy of each node in the neighborhood, we can obtain the direction entropy matrix of the node. Then, the normalized direction entropy matrix and the strength matrix of the node are used to further optimize the strength matrix of the node. Once the node strength matrix is obtained, the salient region in crowd movement can be detected.

## 2. Calculation of Crowd Velocity Vector Field

To calculate the velocity vector field, the crowd video is decomposed into image sequences. Then, each pixel of the image is given a velocity vector calculated using an optical flow algorithm. A motion vector field is thus established. In this paper, considering the spatio-temporal information in motion detection [11], we adopt an improved algorithm based on Lucas-Kanade optical flow algorithm [12] for this task, namely pyramid optical flow algorithm [13].

Lucas-Kanade optical flow, in the process of moving the picture, assumes that a pixel *(x, y)* on the image has a brightness of *I (x, y, t)* at time *t*. After a small time interval of *Δt*, the brightness of the point becomes *I(x + Δx, y + Δy, t + Δt)*. The Taylor formula is used to expand and when *Δt* is small enough to approach zero:

(1) $$I(x+\Delta x,y+\Delta y,t+\Delta t)=I(x,y,t)+\frac{\partial I}{\partial x}\Delta x+\frac{\partial I}{\partial y}\Delta y+\frac{\partial I}{\partial t}\Delta t$$

The optical flow constraint equation can be obtained from the brightness constant:

(2) $$\frac{\partial I}{\partial x}\frac{dx}{dt}+\frac{\partial I}{\partial y}\frac{dy}{dt}+\frac{\partial I}{\partial t}=\frac{\partial I}{\partial x}u+\frac{\partial I}{\partial y}v+\frac{\partial I}{\partial t}=Ixu+Iyv+It=0$$

According to the uniformity of optical flow, we can establish the optical flow equations:

(3) $$\begin{array}{c} Ix1u+Iy1v+It1=0\\ Ix2u+Iy2v+It2=0\\ \vdots \\ Ixnu+Iynv+Itn=0 \end{array}$$

Then use the least square method to gain the Lucas-Kanade optical flow, where *u* is the horizontal velocity and *v* is the vertical velocity:

(4) $$\left[\begin{array}{l} u\\ v \end{array}\right]={\left[\begin{array}{cc} \sum_{i=1}^{n}I_{ix}{}^{2} & \sum_{i=1}^{n}I_{ix}I_{iy}\\ \sum_{i=1}^{n}I_{ix}I_{iy} & \sum_{i=1}^{n}I_{iy}{}^{2} \end{array}\right]}^{-1}\left[\begin{array}{l} -\sum_{i=1}^{n}I_{ix}I_{t}\\ -\sum_{i=1}^{n}I_{iy}I_{t} \end{array}\right]$$

**Figure 2:**
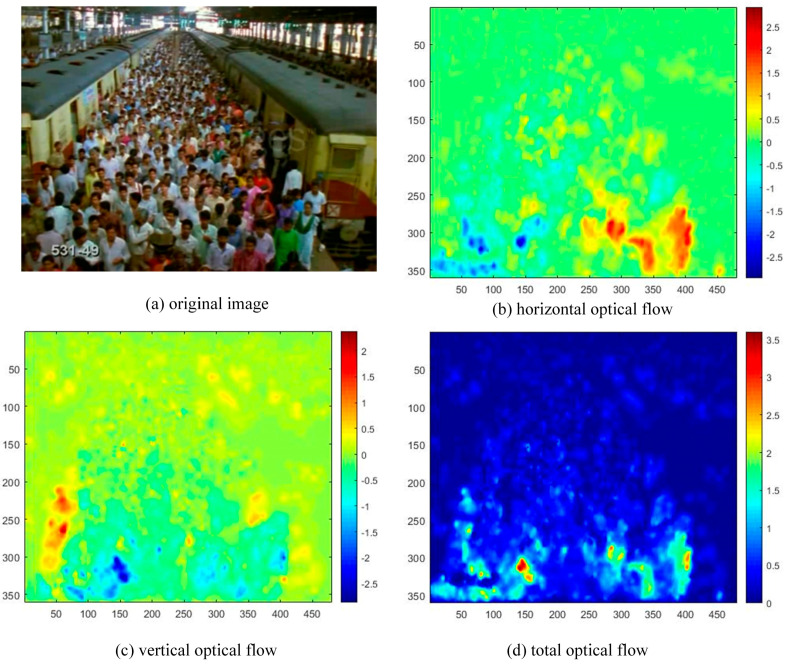
Crowd optical flow field of the sampled frame.

The basic ideas of Lucas Kanade optical flow algorithm are mainly based on three assumptions: (1) constant brightness; (2) time continuous or movement is “small movement”; (3) spatial consistency. If an object is moving fast, the second assumption is not fully satisfied. The value calculated by traditional Lucas-Kanade optical flow will have a larger deviation. Pyramid optical flow algorithm reduces the offset of the target motion by reducing the image layer by layer, which satisfies the hypothesis of optical flow calculation better and weakens the influence of fast target motion. In this paper, the crowd velocity field Q is obtained by the pyramid optical flow algorithm. All the velocity values in the horizontal direction and vertical direction are rounded up. The velocity vector field calculated using pyramid Lucas-Kanade optical flow algorithm for a crowd scene is shown in Figure 2.

## 3. Construction of Repulsive Force Network

### 3.1. Establishment of a Network Node

Complex network is a useful tool for describing a complex system. Each element in the system unit is regarded as a node, and the relationship between elements is regarded as a connection. A complex system can be represented as a network [14]. The crowd velocity vector field can be described as a complex network, in which each velocity vector is a node, and the relationship between the velocity vectors is connected. If the properties of the velocity vectors are measured separately, information stored in the velocity vector cross-correlation cannot be obtained, because the correlation of velocity vectors carries more information than the nature of each velocity [15].

We use the interaction force between particles to construct the network [16,17]. After applying the optical flow method, each vector in the obtained crowd vector field is regarded as a moving microscopic particle. The position and velocity parameters of each particle can be then determined. In our crowd complex network, each particle is treated as a node, and the interaction force between two particles is treated as an edge in the network. Whether there is an edge between the nodes depends on the repulsive force between the nodes, the repulsive force can be described by the inertial centrifugal force, and the value of the inertial centrifugal force is the weight of the edge. A weighted undirected network *G^w^* node set $Q=\{q_{1},q_{2},\cdots ,q_{n}\}$ can be generated, where *n* is the total number of nodes. The number of network nodes is equal to the number of particles in the crowd velocity field.

### 3.2. Establishing the Network Edges Using Repulsive Force Model 

In the whole particle field, the size and direction of particle velocity are instantaneous, and the motion of the next times is random. If the moving particle is assumed as an agent, there is a possibility of interaction and collision between particles in motion. Imagine that each particle is an agent. In order to avoid collision between agents, an agent adds a repulsive force element to prevent them from colliding with each other. This repulsive force can be described by inertial centrifugal force [18]. For a given crowds particle field *Q (M, N)* in the column *N* and row *M*, selecting a particle $q_{xo}{}_{yo}$ as the node, constructing a two-dimensional neighborhood *δ*, the size is $\left(x_{0}\pm \varepsilon ,y_{0}\pm \varepsilon \right)$. In this region, the connection between $q_{xo}{}_{yo}$ and other nodes $q_{xy}$$\left(x\neq x_{0},y\neq y_{0}\right)$ can be described as $e(q_{xoyo},q_{xy})$. Whether this connection exists is determined by the following formula:

(5) $$e(q_{xoyo},q_{xy})\{\begin{array}{cc} \exists , & {\vec{F}}_{ij}\neq 0,q_{xy}\in \delta \\ ∄, & \mathrm{otherwise} \end{array}$$

The formula for calculating the inertial centrifugal force is as follow:

(6) $${\vec{F}}_{ij}=-m_{i}k_{ij}\frac{v_{ij}^{2}}{dist_{ij}}{\vec{e}}_{ij}$$

where ${\vec{e}}_{ij}$ is the direction vector and $m_{i}$ is the mass of particle $q_{i}$. In this paper, the mass of all particles is set as unit 1.$v_{ij}$ is the relative velocity of two particles, $dist_{ij}$ is the distance between two particles, $k_{ij}$ is a coefficient, the calculation of $v_{ij}$ and $k_{ij}$ is determined by the following formula:

(7) $$v_{ij}=\left\{ \begin{array}{ll} ({\vec{v}}_{i}-{\vec{v}}_{j})\cdot {\vec{e}}_{ij}, & ({\vec{v}}_{i}-{\vec{v}}_{j})\cdot {\vec{e}}_{ij}>0\\ 0, & \mathrm{others} \end{array}\right.$$

(8) $$k_{ij}==\left\{ \begin{array}{ll} ({\vec{v}}_{i}\cdot {\vec{e}}_{ij})/v_{i}, & {\vec{v}}_{i}\cdot {\vec{e}}_{ij}>0,v_{i}\neq 0\\ 0, & \mathrm{others} \end{array}\right.$$

Then, we can obtain the joint weight, which can be expressed by the magnitude of the repulsive force:

(9) $$We=\left|{\vec{F}}_{ij}\right|$$

**Figure 3:**
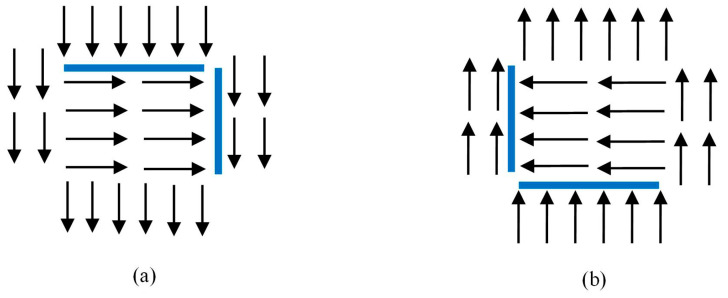
Schematic diagram of repulsive force: (**a**) original motion flow; (**b**) motion flow reversed.

According to the repulsive force formula, if the particle moves away from the affected particle, the repulsive force will be very low. As shown in Figure 3, the arrow represents the moving optical flow, and the blue line represents the repulsive force generated. Figure 3a is the schematic diagram of the repulsive force in the original direction, and Figure 3b is the schematic diagram of the repulsive force in the opposite direction. Thus, for some application of salient region detection, only half of the boundary can be detected. 

**Figure 4:**
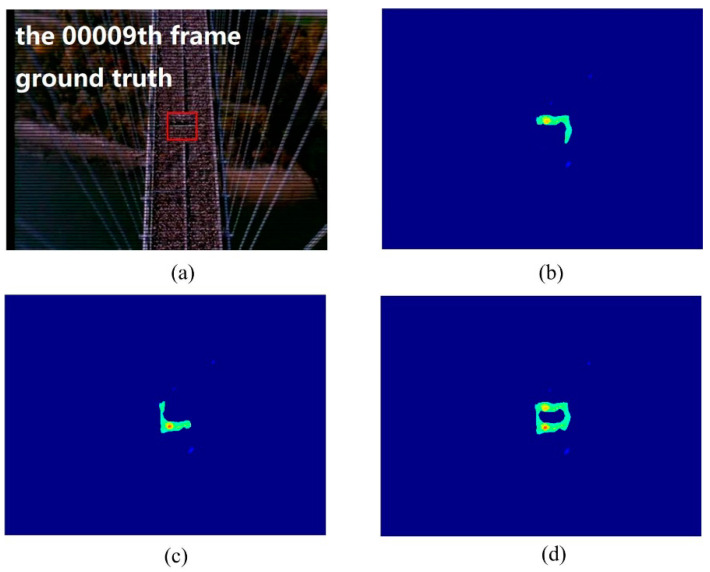
Expressing the effect of optical flow reversal: (**a**) original sample frame; (**b**) detection result using original optical flow; (**c**) detection result using reversed optical flow; (**d**) detection result after the integration of optical flow.

In order to get a complete boundary, the velocity field is reversed and the repulsive force between particles is calculated repeatedly. Thus, the repulsive force model can be used to construct the edge and weight of the repulsive force network. The results of the superposition of the two are taken as a construction step. Figure 4 shows an example. If we construct the repulsive force network for the optical flow field of the original video sequence, only half of the boundary can be obtained. If we construct the repulsive force network again after reversing the optical flow field, the other half of the boundary can be obtained. 

The two-dimensional crowd velocity field is transformed into weighted undirected network model $G^{w}(Q,E,We)$ by repeating the above steps for each node. The corresponding weighted undirected network node is set as $Q=\{q_{1},q_{2},\cdots ,q_{n}\}$ and the network edge is set as $E=\left\{e_{1},e_{2},\cdots ,e_{m}\right\}$. In the crowds weighted network model, the connection between nodes and the degree of connection between nodes can be expressed by the following adjacency matrix:

(10) $$A=\left[\begin{array}{cccc} \left|\vec{F_{11}}\right| & \left|\vec{F_{12}}\right| & \ldots & \left|\vec{F_{1n}}\right|\\ \vdots & \vdots & \ddots & \vdots \\ \left|\vec{F_{n1}}\right| & \left|\vec{F_{n2}}\right| & \cdots & \left|\vec{F_{nn}}\right| \end{array}\right],$$

### 3.3. Calculation of Node Strength

Statistical characteristic parameters of a network can be used to represent the characteristics of a network, such as node degree, average path length, clustering coefficient. In this paper, node strength is chosen to describe the characteristics of the crowd complex network. In the complex network model, node strength is the generalization of node degree, which integrates the strength between edges and nodes [19,20]. From the adjacency matrix, the node strength $s(q_{i})$ of node $q_{i}$ can be expressed as follows:

(11) $$s(q_{i})=\sum_{n=1}^{j}\left|\vec{F_{ij}}\right|,$$

After calculating each point in the crowd velocity field, we can get the node strength of all nodes. The node strength field *S(M, N)* is also a two-dimensional matrix containing *M* rows and *N* columns. There is also a one-to-one correspondence between the node strength field and the crowd speed field:

(12) $$S=\left[\begin{array}{cccc} S_{11} & S_{12} & \ldots & S_{1N}\\ \vdots & \vdots & \ddots & \vdots \\ S_{M1} & S_{M2} & \cdots & S_{MN} \end{array}\right],$$

In order to facilitate the node strength field optimization operation in later stage, the node strength field is normalized as follows:

(13) $$S\prime =\frac{S-S_{min}}{S_{max}-S_{min}}\left[\begin{array}{cccc} S_{11} & S_{12} & \ldots & S_{1N}\\ \vdots & \vdots & \ddots & \vdots \\ S_{M1} & S_{M2} & \cdots & S_{MN} \end{array}\right],$$

where $S_{max}$ and $S_{min}$ are the maximum and minimum values of the nodes in all node strengths.

## 4. Optimizing Node Strength Field Using Direction Entropy

### 4.1. Establishment of Vector Direction Entropy Matrix

For a crowd motion field *Q (M, N)* of the *M* row and *N* column, one particle $q_{xo}{}_{yo}$ is selected, and thus, the direction angle of particle motion is divided into eight directions at 45 degrees interval. The calculation of velocity direction angle and direction grade is determined by the following formula:

(14) $$\theta =\arctan\frac{q_{\mathrm{yo}}}{q_{xo}},$$

(15) $$d=\{\begin{array}{cc} 1 & 0\leq \theta <\frac{\pi }{4}\\ \vdots & \vdots \\ 8 & \frac{7\pi }{4}\leq \theta <2\pi \end{array},$$

Choose a two-dimensional neighborhood *δ* with the same edge and weight as the repulsive force model with the size of $\left(x_{0}\pm \varepsilon ,y_{0}\pm \varepsilon \right)$. For a sub-image region, because of the different motion forms of particles, the direction of particle motion is uncertain at eight angles. Shannon entropy is a classical method to measure the uncertainty of information, and is the basis of communication science [21,22,23]. In this paper, Shannon entropy is used to measure the uncertainty of particle motion direction. In this paper, we employ Shannon entropy to describe the chaotic degree of crowd motion. In a neighborhood *δ*, each particle can be calculated by direction rank formula to get a direction rank *d*. Each direction rank occupies a certain probability *p_i_* in all direction ranks. According to the definition of Shannon entropy [21] and [23], we can assign the velocity direction entropy between the central particle $q_{xo}{}_{yo}$ and other particles $q_{xy}$$\left(x\neq x_{0},y\neq y_{0}\right)$ neighboring the central particle. The calculation is determined by the following formula:

(16) $$H_{xoyo}=-\sum_{i=1}^{n}p_{i}\log p_{i},\;n=\varepsilon^{2},$$

For each position, in the crowd particle field *Q* (*M, N*), the entropy can be calculated by repeating the steps mentioned above. Therefore, the direction entropy of each particle in the crowd particle field can be obtained. The two-dimensional crowd velocity vector field can be transformed into a particle direction entropy matrix:

(17) $$H=\left[\begin{array}{cccc} H_{11} & H_{12} & \ldots & H_{1N}\\ \vdots & \vdots & \ddots & \vdots \\ H_{M1} & H_{M2} & \cdots & H_{MN} \end{array}\right],$$

where, H*_11_, H_12_……H_MN_* is the entropy at the corresponding position of the crowd particle field. In order to facilitate the node strength field optimization operation in later stage, the direction entropy matrix is normalized as follows:

(18) $$H\prime =\frac{H-H_{min}}{H_{max}-H_{min}}\left[\begin{array}{cccc} H_{11} & H_{12} & \ldots & H_{1N}\\ \vdots & \vdots & \ddots & \vdots \\ H_{M1} & H_{M2} & \cdots & H_{MN} \end{array}\right],$$

$H_{max}$ and $H_{min}$ are the maximum and minimum values in the entropy matrix for all directions.

### 4.2. Optimizing the Node Strength Field

The direction entropy matrix of crowd movement can describe the degree of changes in the direction of movement of the nodes. Furthermore, the strength field of the repulsive force node describes the degree of repulsion of each node and the surrounding nodes. In order to reduce the noise caused by other interference motion, this paper combines these two kinds of model to optimize the node strength field. It is very important to choose an effective way to integrate these two features, e.g., node strength and entropy. There are many ways to integrate features, such as multiplication and addition. For the application of salient crowd motion detection, the way of feature fusion requires significant expression of specific crowd motion regions and adaptation to the changes of scene. We analyzed the feature of node strength and entropy. The saliency region can be detected by combining the two features by multiplying or add. However, the saliency region obtained by addition is more effective. Because the range of the two features is quite different and there are great changes in different scenarios, it is difficult to determine the combined weights. Therefore, this paper applies a normalized processing of the two features before adding the two features together. Although there are differences in dimension between them, as a normalized feature, it works well when integrating them at the application level.

The direction entropy matrix of crowd motion is in one-to-one correspondence with the strength field of nodes; thus, we have made a comparison according to the following formulas:

(19) $$P_{ij}=\{\begin{array}{cc} S_{ij}{}^{\prime }+H_{ij}{\;}^{\prime } & S_{ij}{}^{\prime }\neq 0,H_{ij}{}^{\prime }\neq 0\\ 0 & \mathrm{others} \end{array},$$

The optimized node strength field is:

(20) $$P=\left[\begin{array}{cccc} P_{11} & P_{12} & \ldots & P_{1N}\\ \vdots & \vdots & \ddots & \vdots \\ P_{M1} & P_{M2} & \cdots & P_{MN} \end{array}\right],$$

Then, for nomalizing the optimized node strength field, the specific calculation formula is as follows:

(21) $$P\prime =\frac{P-P_{min}}{P_{max}-P_{min}}\left[\begin{array}{cccc} P_{11} & P_{12} & \ldots & P_{1N}\\ \vdots & \vdots & \ddots & \vdots \\ P_{M1} & P_{M2} & \cdots & P_{MN} \end{array}\right],$$

After normalizing the strength field of the nodes, we smoothed the node strength field with a 3 × 3 mean filter template. It can eliminate the negative effects of the node strength caused by too high or too low values on the experimental results. In order to intuitively describe and observe the value of node strength, we use a pseudo-color image display method to visualize node strength. Pseudo-color image shows the pixel value corresponding to the node strength value. In a crowd scene, it is obvious that the node pixel values in salient regions are higher than those in other regions.

## 5. Experimental Results and Analysis

In our experiments, we tested three crowded scene video sequences from Crowd Saliency dataset [5] and a video sequence in [24] to show the performance of the proposed method. Retrograde and instability regions of a crowd were detected in the experiment. For different crowded scenes, the scale of the velocity field *Q(M,N)* and the parameters *ε* (the size of neighborhood) in the experiment are shown in Table 1. The proposed method is effective for images used in this experiment, which do not have a high resolution. If it is used to deal with high resolution images, there are two ways to processing the data. One is to reduce the high-resolution image using interval sampling and local mean, and the other is to process optical flow data by interval sampling. 

### 5.1. Crowd Retrograde Behavior Detection

**Figure 5:**
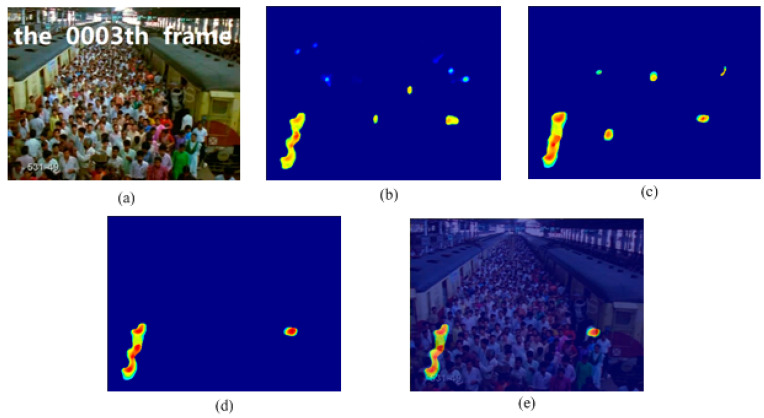
Retrograde motion detection in train station scene: (**a**) input frame; (**b**) node strength field of repulsive force network; (**c**) detection result using direction entropy; (**d**) salient region detection after optimized; (**e**) overlap the salient region with input frame.

**Figure 6:**
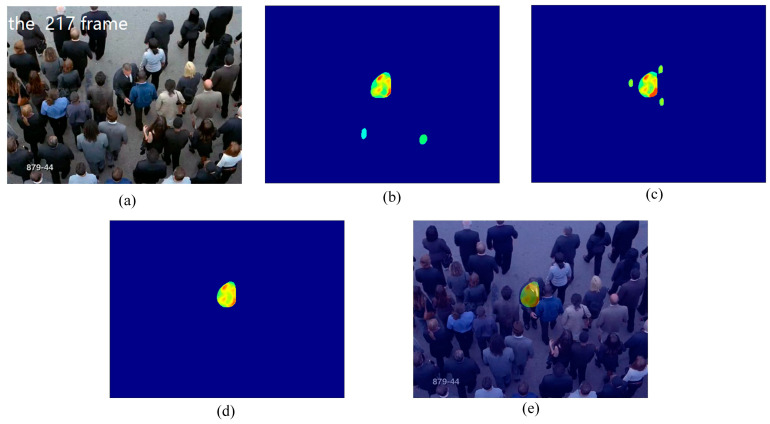
Retrograde motion detection in single retrograde scene: (**a**) input frame; (**b**) node strength field of repulsive force network; (**c**) detection result using direction entropy; (**d**) salient region detection after optimized; (**e**) overlap the salient region with input frame.

In this experiment, we used the train station scene and the single retrograde scene to show the salient detection for retrograde behavior. As shown in Figure 5 and Figure 6, some pedestrians do not conform to the flow of the mainstream crowd, hence, a retrograde motion was formed instead. The particles will thus have a larger repulsion force and direction entropy in this region. This proposed method can effectively detect human retrograde movement. Figure 5a and Figure 6a shows the original video frame. The node strength field calculated from the repulsive force network is shown in Figure 5b and Figure 6b. It can be clearly seen that the regions with high node strength represents the retrograde motion. However, there are still some disturbances. As shown in Figure 5c and Figure 6c, though the entropy value of the retrograde region is large, there are still some noise regions. Fortunately, the disturbance regions detected by node strength and direction entropy are different. Therefore, we can optimize the saliency detection results by integrating node strength and direction entropy, as is shown in Figure 5d and Figure 6d. In order to illustrate the detection performance, we overlap the saliency detection results with the original video frames in Figure 5e and Figure 6e. Experiments show that our method can detect pedestrians who even move oppositely to the flow of mainstream crowd.

### 5.2. Crowd Motion Instability Region Detection

**Figure 7:**
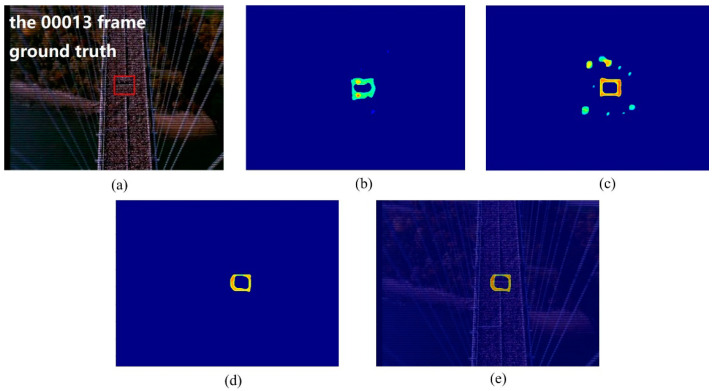
Salient crowd instability motion detection in marathon scene: (**a**) original video frame and ground true (red box); (**b**) node strength field of repulsive force network; (**c**) detection result using direction entropy; (**d**) salient region detection after optimized; (**e**) overlap the salient region with original video frame.

**Figure 8:**
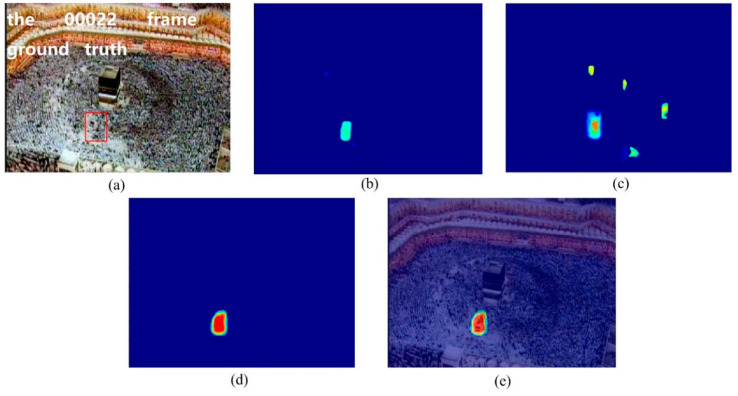
Salient crowd instability motion detection in pilgrimage scene: (**a**) original video frame and ground true (red box); (**b**) node strength field of repulsive force network; (**c**) detection result using direction entropy; (**d**) salient region detection after optimized; (**e**) overlap the salient region with original video frame.

In the crowd surveillance system, the instability area of crowd movement often deserves attention. In this experiment, we used two scenes, including the marathon scene (Figure 7) and the pilgrimage scene (Figure 8) to show the performance of the proposed method for detecting the instability crowd motion. 

The sample frames for the two scenarios are shown in Figure 7a and Figure 8a. There are instability motion regions (some pedestrians are different from the mainstream crowd) in these two crowds. The results of node strength fields of two scenes are shown in Figure 7b and Figure 8b, respectively. We can see that the node strength of the repulsive force model is larger in instability motion regions. The direction entropy fields of two scenarios are shown in Figure 7c and Figure 8c, respectively. The entropy values of the instability region are clearly large. However, there is some noise in the unstable region detected by any single method. After integrating these two methods of node strength and direction entropy, the saliency detection results are optimized and the interference areas are effectively removed, which can be seen in Figure 7d and Figure 8d. Figure 7e and Figure 8e show saliency detection results after overlapping with the original video frame. Experimental results show that the proposed method can detect the salient crowd instability motion in large-scale crowded scenes.

### 5.3. Detection Results Using Different Neighborhood Size 

**Figure 9:**
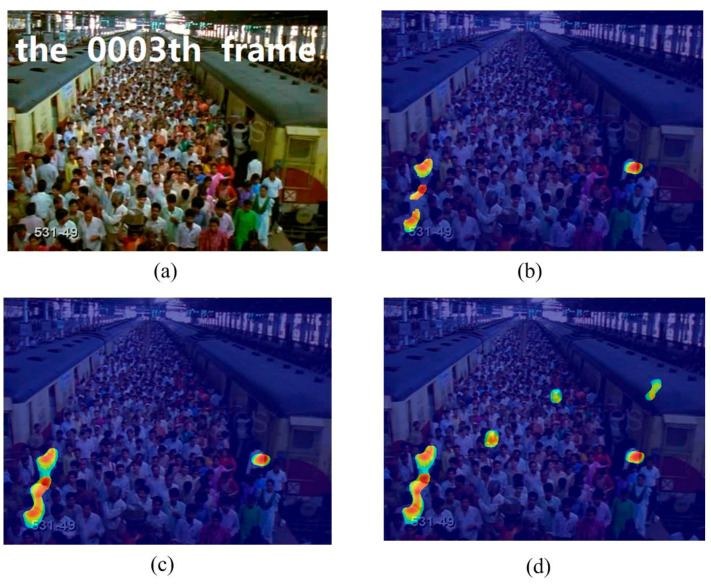
Retrograde motion detection in train station scene using different neighborhood size: (**a**) original video frame; (**b**) detection result using 5 × 5 neighborhood; (**c**) detection result using 13 × 13 neighborhood; (**d**) detection result using 23 × 23 neighborhood.

**Figure 10:**
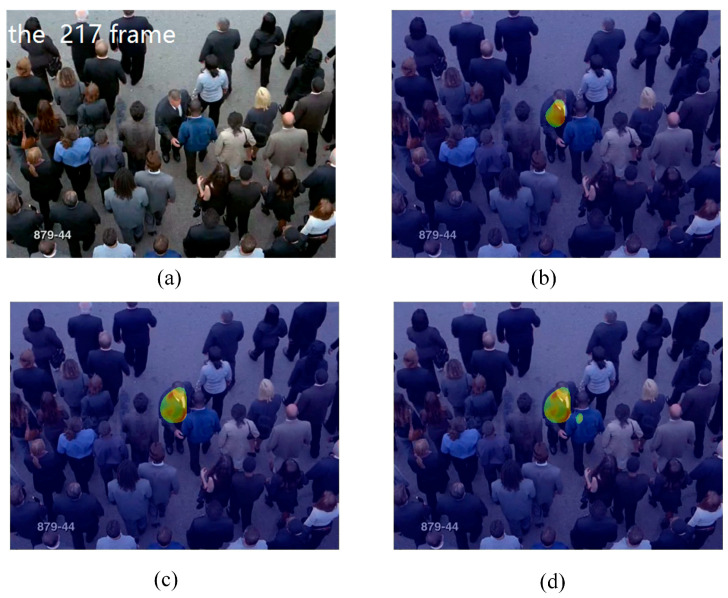
Retrograde motion detection in single retrograde scene using different neighborhood size: (**a**) original video frame; (**b**) detection result using 5 × 5 neighborhood; (**c**) detection result using 15 × 15 neighborhood; (**d**) detection result using 23 × 23 neighborhood.

**Figure 11:**
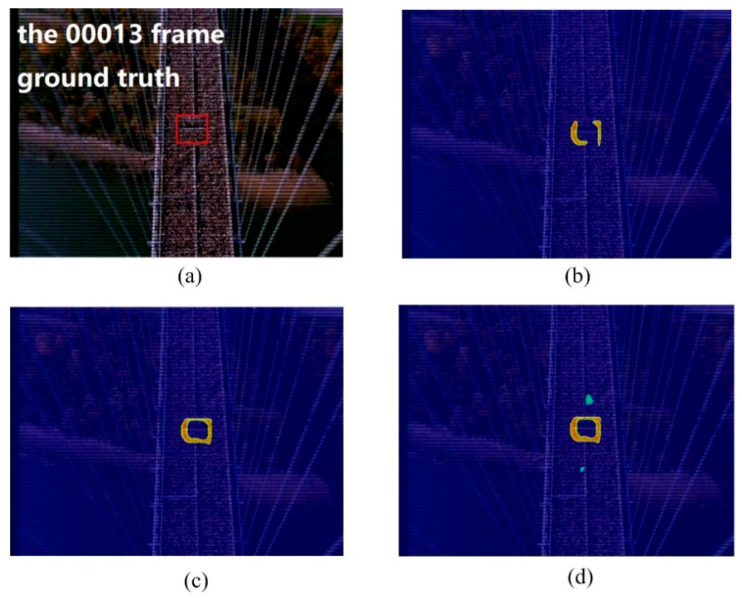
Instability motion detection in marathon scene using different neighborhood size: (**a**) original video frame and ground truth region; (**b**) detection result using 5 × 5 neighborhood; (**c**) detection result using 11 × 11 neighborhood; (**d**) detection result using 23 × 23 neighborhood.

**Figure 12:**
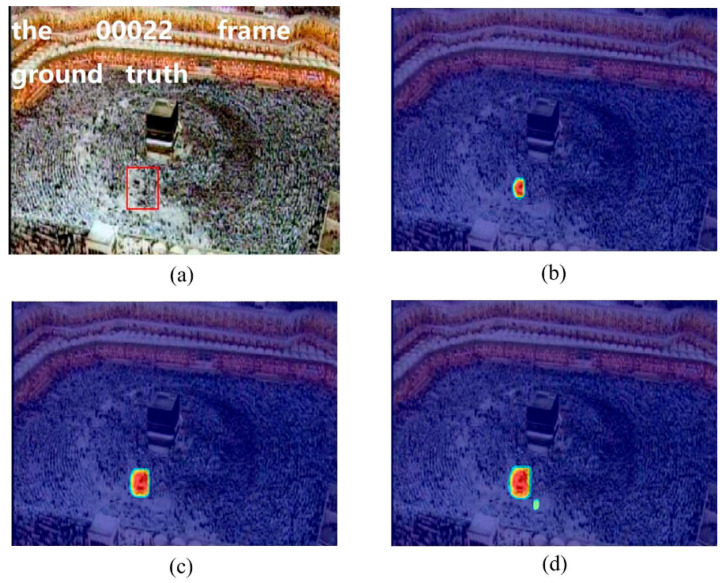
Instability motion detection in pilgrimage scene using different neighborhood size: (**a**) original video frame and ground truth region; (**b**) detection result using 5 × 5 neighborhood; (**c**) detection result using 15 × 15 neighborhood; (**d**) detection result using 25 × 25 neighborhood.

It is very important to select a suitable neighborhood size *ε* to construct complex network. A neighborhood that is too small will not be bias to salient motion, while a neighborhood with too large a scale will introduce more noise. In this section, the salient crowd motion will be detected using different neighborhood sizes *ε*. For retrograde motion detection, the train station scene and single retrograde scene is used to show the performance of the proposed method. From Figure 9, we can see that the salient motion region detected by applying the size of 5 × 5 neighborhood is slightly scattering, while the area detected by the size of 13 × 13 is more complete. A larger the neighborhood 23 × 23, can cause more noise in the detection results. As for the Figure 10, the salient motion region size detected by using the 5 × 5 neighborhood is small, while the area detected by using the 15 × 15 neighborhood size is more complete. When choosing a larger neighborhood of 23 × 23, the result includes more noise. For instability motion detection, two scenes were used in this experiment. For the marathon scene, the detection result was usually not closed if the neighborhood size was too small (5 × 5 neighborhood). Applying a neighborhood size of 23 × 23, noise interference will be introduced, although closed salient motion regions can still be obtained. After the experiment, the closed salient motion region can be obtained using a neighborhood size of 11 × 11 (Figure 11). For another pilgrimage scene, although the saliency region can also be detected with a 5 × 5 size neighborhood, the result obtained with a 15 × 15 size neighborhood is closer to the ground truth (Figure 12).

### 5.4. Performance Evaluation and Comparison

The ground truth of crowd salient detection for pilgrimage and marathon scene has been given in the Crowd Saliency dataset [5]. The ground truth is given using a rectangular area. In order to evaluate the performance of the proposed method, we calculate the minimum enclosing rectangle of the detected salient motion region. To quantitatively evaluate the performance of the method, two indicators (precision and recall) are calculated in our experiments. In this paper, precision is the ratio of the number of pixels in the detected region that belong to the ground truth to the number of pixels in the detected area, indicating whether the number of pixels in the detected local motion instability area is accurate, expressed by *Pr*. Recall is the ratio of the number of pixels in the detected results region that belonging to the instability motion region to the number of all pixels of the ground truth, represented by *R* [25]. The precision and recall can be calculated as:

(22) $$Pr=\frac{TP}{TP+FP}$$

(23) $$R=\frac{TP}{TP+FN}$$

where *TP* indicates that both the detection result and the ground truth are positive. *FP* indicates that the detection is positive and the actual is negative. *TN* indicates that both the prediction and the ground truth are negative. *FN* indicates that the prediction is negative but the actual is positive. 

**Figure 13:**
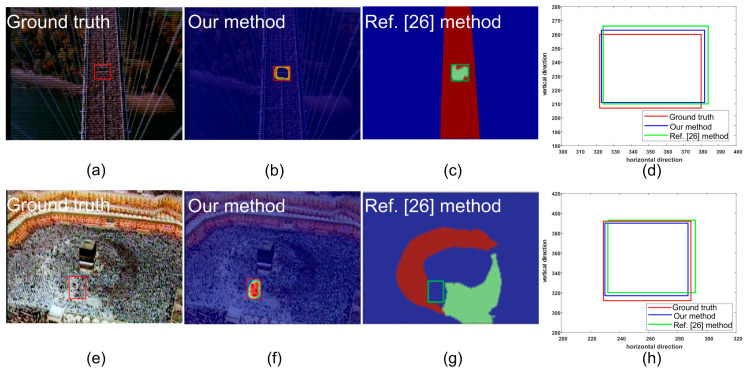
Comparison of the method in this paper with the article [26]: (**a**,**e**) are the ground truth of marathon and pilgrimage scene; (**b**,**f**) are the results gained by our method; (**c**,**g**) are courtesy of reference [26]; (**d**,**h**) are the local enlarged displays of the results.

The precision and recall calculated from the pilgrimage and marathon scene using different parameters (the size of neighborhood) are given in Table 2. Obviously, according to the parameters selected in this paper, satisfactory detection accuracy can be obtained. If the neighborhood size is too large or too small, the detection accuracy will be seriously affected. Figure 13 shows the detection results of the pilgrimage and marathon scene using different methods. From Figure 13 we can see that both the proposed method and the methods mentioned in [26] can detect the salient region correctly. However, the rectangular region obtained by the proposed method is closer to the ground truth. 

## 6. Conclusions

In this paper, we proposed a method for crowd salient motion detection based on a direction entropy and a repulsive force network. This paper focused on how to detect saliency regions in crowd movement effectively. Firstly, the crowd video sequence frames are processed by the optical flow algorithm followed by the crowd velocity vector field calculation. Secondly, according to the repulsive force model, the interaction force between two particles is determined as a certain condition. The repulsive force network is obtained and the strength of the crowd weighted network node is extracted as the characteristic parameter to construct a two-dimensional feature matrix. Finally, the velocity vector direction entropy is combined with the repulsive force network characteristic matrix to detect the salient crowd motion structure. The experimental results of four crowd video sequences show that the proposed method can not only detect the region of retrograde behavior of crowd movement but also the region of unstable crowd movement in large-scale crowd scenes. For future work, we will focus on the development of a method for an adaptive threshold and neighborhood calculation.

## Figures and Tables

**Table 1:** Different scenes and parameter values.

Crowded Scenes	Symbol of Parameter	The Value
Train station scene in Figure 5	*ε* *M × N*	13 480 × 360
Single retrograde scene in Figure 6	*ε* *M × N*	15 480 × 360
Marathon scene in Figure 7	*ε* *M × N*	11 640 × 480
Pilgrimage scene in Figure 8	*ε* *M × N*	15 640 × 480

**Table 2:** The measurement of the accuracy of the detection results using different parameters.

Crowded Scenes	Statistics	Size of Neighborhood	Results
**marathon**	*Pr*	5 × 5	0.862
		11 × 11	0.910
		23 × 23	0.531
	*R*	5 × 5	0.841
		11 × 11	0.909
		23 × 23	0.877
**pilgrimage**	*Pr*	5 × 5	1
		15 × 15	1
		25 × 25	0.684
	*R*	5 × 5	0.244
		15 × 15	0.867
		25 × 25	0.656
